# Chiral Jahn–Teller Distortion in Quasi-Planar Boron Clusters

**DOI:** 10.3390/molecules29071624

**Published:** 2024-04-04

**Authors:** Dongbo Zhao, Yilin Zhao, Tianlv Xu, Xin He, Shankai Hu, Paul W. Ayers, Shubin Liu

**Affiliations:** 1 Institute of Biomedical Research, Yunnan University, Kunming 650500, China; 2 Department of Chemistry and Chemical Biology, McMaster University, Hamilton, ON L8S 4M1, Canada; 3 College of Chemistry and Chemical Engineering, Hunan Normal University, Changsha 410081, China; 4 Qingdao Institute for Theoretical and Computational Sciences, Shandong University, Qingdao 266237, China; 5 Research Computing Center, University of North Carolina, Chapel Hill, NC 27599-3420, USA; 6 Department of Chemistry, University of North Carolina, Chapel Hill, NC 27599-3290, USA

**Keywords:** Jahn–Teller effect, helical spin density, helical molecular orbital, boron clusters

## Abstract

In this work, we have observed that some chiral boron clusters ($B_{16}^{-}$, $B_{20}^{-}$, $B_{24}^{-}$, and $B_{28}^{-}$) can simultaneously have helical molecular orbitals and helical spin densities; these seem to be the first compounds discovered to have this intriguing property. We show that chiral Jahn–Teller distortion of quasi-planar boron clusters drives the formation of the helical molecular spin densities in these clusters and show that elongation/enhancement in helical molecular orbitals can be achieved by simply adding more building blocks via a linker. Aromaticity of these boron clusters is discussed. Chiral boron clusters may find potential applications in spintronics, such as molecular magnets.

## 1. Introduction

While they are not experimental observables, molecular orbitals are a conceptually useful and elegant tool for elucidating molecular properties [1]. They have long been a significant tool in the arsenal of chemists [2,3,4,5,6], tracing back to the early work of Hückel, Mulliken, and others. One prominent example is the principle of conservation of orbital symmetry [7,8,9], which subsumes the Woodward–Hoffmann rules. Helical frontier molecular orbitals, first introduced by Hendon et al. [10] in 2013, have seeded a surge of interest. Helical frontier molecular orbitals appear in disubstituted allenes and even-*n* cumulenes. Later, many more types of molecules possessing this interesting property were reported [11,12,13,14,15,16,17,18,19,20,21,22,23,24,25,26,27,28,29,30], and it was discovered that molecules with helical orbitals have interesting physicochemical properties. For example, oligoyne-bridged bifluorenes can induce spin–orbit coupling [30].

**Scheme 1:**
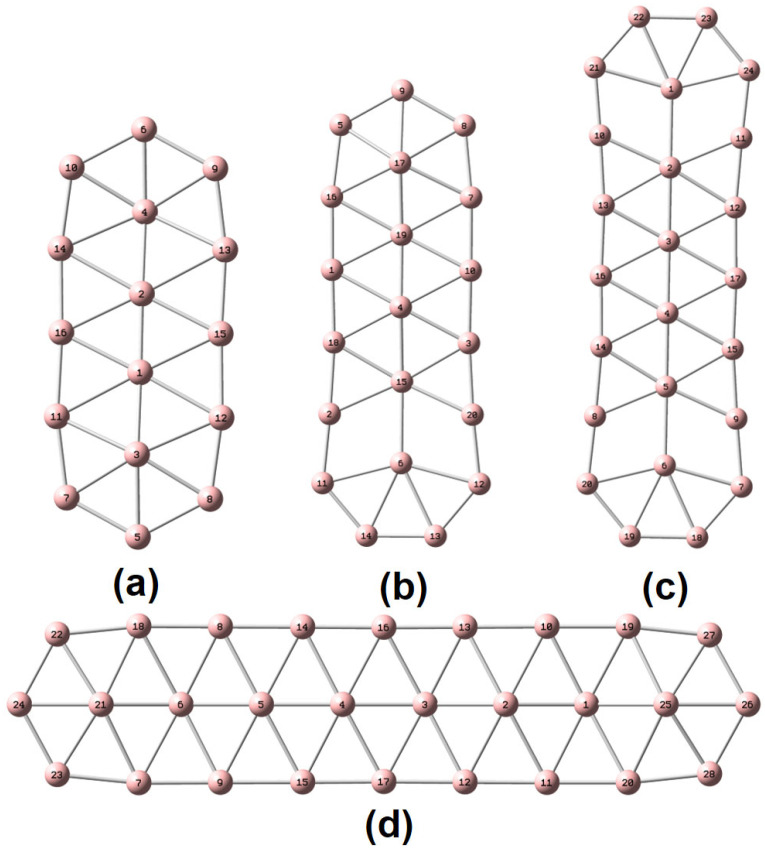
Molecular representation of (**a**) $B_{16}^{-}$, (**b**) $B_{20}^{-}$ (**c**) $B_{24}^{-}$ and (**d**) $B_{28}^{-}$ and the corresponding atomic numberings.

Boron forms clusters with unique bonding, aromaticity, and reactivity properties [31,32,33]. Very recently [34], for the first time, we observed helical spin densities of anionic boron clusters. In this work, we report that $B_{16}^{-}$ [35], $B_{20}^{-}$ [36], $B_{24}^{-}$ [37], and $B_{28}^{-}$ [38] (see Scheme 1) not only have helical molecular orbitals, but also helical spin densities. This is interesting because, unlike molecular orbitals, spin densities are experimental observables, and this allows the edifice of (spin)-resolved (conceptual) density-functional theory [39,40,41,42,43,44,45,46,47,48,49,50] to be directly applied to these compounds.

To pin down the origin of spin-density helicity, we forced the quasi-planar boron atoms to be exactly in a plane (where we simply set a column of Cartesian coordinates to zero): helical spin densities are no longer observed. Thus, it is chiral Jahn–Teller distortion that governs the formation of helical spin densities. This seems to be the first observation of this intriguing phenomenon in inorganic boron clusters. We also show that the elongation or enlargement of helical molecular orbitals can be achieved by simply adding more structural motifs via a linker. Moreover, we have exhibited that helical-shape molecules have a large propensity to assume helical molecular orbitals as shown in inorganic species $P_{9}^{+}$ [51], Be_6_$B_{11}^{-}$ [52], and ${\mathrm{As}}_{11}^{3-}$ [53] (vide infra).

## 2. Results

**Figure 1:**
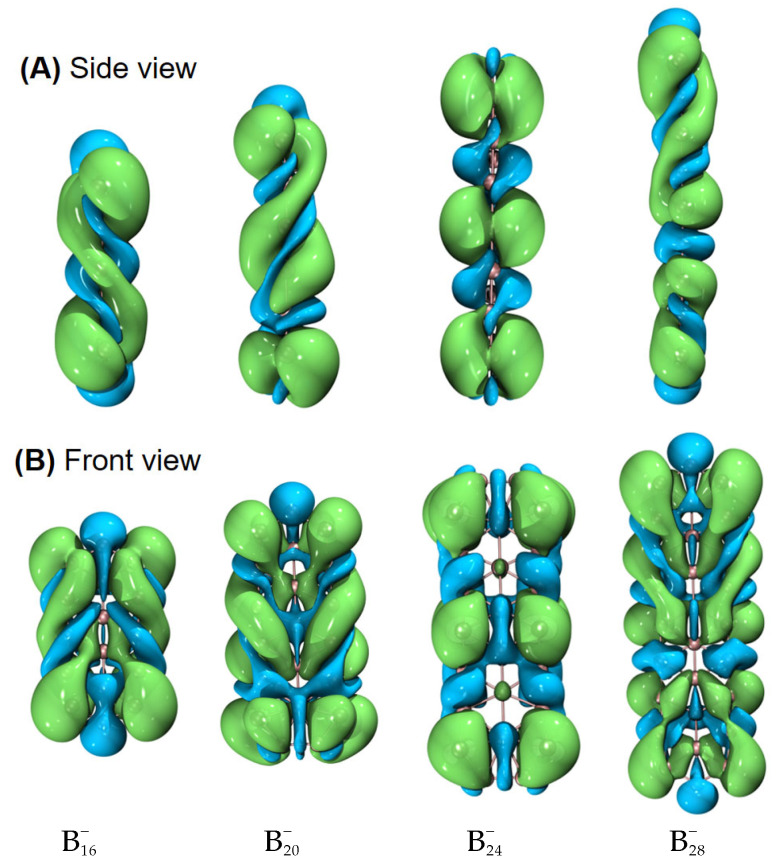
Helical spin densities of quasi-planar boron clusters $B_{16}^{-}$, $B_{20}^{-}$, $B_{24}^{-}$, and $B_{28}^{-}$ obtained at the PBE0/6-311+G(d) level from both (**A**) side and (**B**) front views. The isovalue was set to be 0.0004 a.u. Molecular renderings were achieved via the VMD 1.9.4 [55] software.

To characterize the planarity of anionic boron clusters (in both the ground and excited state), we used a few parameters [54], including the molecular planarity parameter (MPP), span of deviation from the plane (SDP), and maximum positive/negative deviation (MPD/MND) to the fitted plane, as listed in Table 1. The definitions of MPP, SDP, MPD, and MND are given in Section 4. The fitted parameters of a plane are listed in Table S1. Of note, in this work, the planarity of a molecule is a geometric concept. Exact planarity means that all the atoms lie in a plane, simply like a benzene molecule. Quasi-planarity indicates that one or more than one atom lie slightly above/below a plane. One can easily discover that all the systems studied in this work are quasi-planar. Based upon the optimized structures in the ground state, we have observed helical spin densities as exhibited in Figure 1. No similar results are discerned for the excited-state structures (see Figure S1).

**Figure 2:**
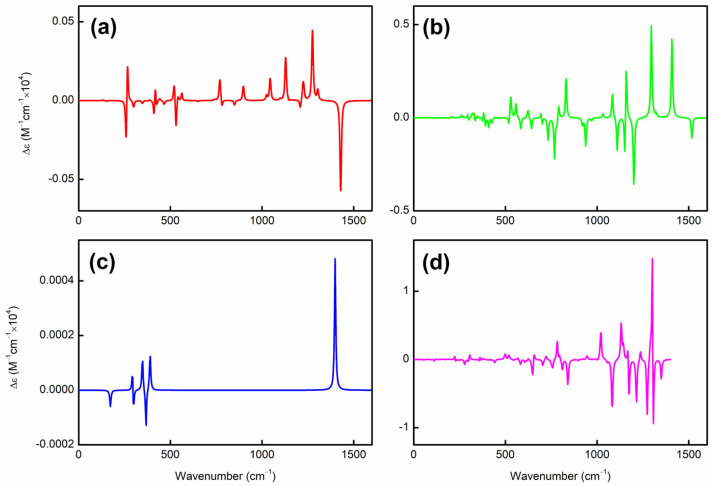
VCD spectra of (**a**) **$B_{16}^{-}$**, (**b**) **$B_{20}^{-}$**, (**c**) **$B_{24}^{-}$**, and (**d**) **$B_{28}^{-}$** obtained at the PBE0/6-311+G(d) level. The *x*/*y*-axis spans from (**a**) to (**d**).

**Figure 3:**
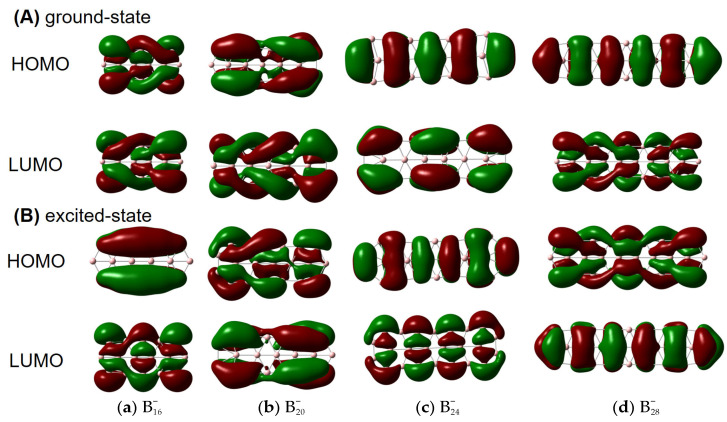
*β*–HOMOs and *β*–LUMOs of (**A**) ground- and (**B**) excited-state **$B_{16}^{-}$**, **$B_{20}^{-}$**, **$B_{24}^{-}$**, and **$B_{28}^{-}$** obtained at the PBE0/6-311+G(d) level from both side and front views. The isovalue was set to be 0.02 a.u. Molecular renderings were achieved via the GaussView 6.0 [58] software.

To elucidate the origin of helical spin densities, we force the ground-state quasi-planar boron structures to be exactly planar followed by single-point calculations at the PBE0/6-311+G(d) [56,57] level. The spin densities’ helicity then vanishes (see Figure S2). Accordingly, a chiral Jahn–Teller distortion plays a key role where the right- and left-handed deformations are (quasi)equal in energy, and the planar structure deforms slightly to break symmetry, thus lowering in energy. More intriguingly, these chiral structures [in terms of vibrational circular dichroism (VCD) spectra; see Figure 2 for details] can also have helical frontier molecular orbitals as shown in Figure 3. Of note, excited-state VCD spectra are also observed as shown in Figure S3. In the ground state, $B_{16}^{-}$, $B_{20}^{-}$ and $B_{28}^{-}$ (but not $B_{24}^{-}$) have helical *β*–LUMOs (with lower orbital energies than their *α* counterparts) and $B_{16}^{-}$ and $B_{20}^{-}$ also have helical *β*–HOMOs in Figure 3; in the excited state, only $B_{20}^{-}$ and $B_{28}^{-}$ have helical *β*–HOMOs. Then, how to understand such a phenomenon?

Our results seem to be specific to π-electron deficient (Be, B) and abundant (P, As) structures, where buckling evidently occurs to accommodate a slight contamination with chirality-supporting sp^3^ hybridization. Presumably the difference in these different clusters comes from a delicate balance between a preference for Hückel-esque orbitals and chiral orbitals, which in turn probably reflects electron correlation. We note that the boron cluster with achiral HOMO and LUMO ($B_{28}^{-}$) is the least strongly correlated, suggesting that near-degeneracy of the valence orbitals is important for induced chirality. More specifically, the chirality seems to be induced by the type of strong electron correlation that can be modelled with spin-symmetry breaking, which is reflected in the fact that the *α* and *β* HOMO and HOMO-1 energies differ quite significantly in $B_{16}^{-}$ and $B_{20}^{-}$, but much less in $B_{24}^{-}$. In addition, $B_{24}^{-}$ has a remarkably large gap between the low-energy *β*-LUMO and the higher-energy *α*-LUMO, which perhaps explains its exceptional behavior in Figure 3A.

**Figure 4:**
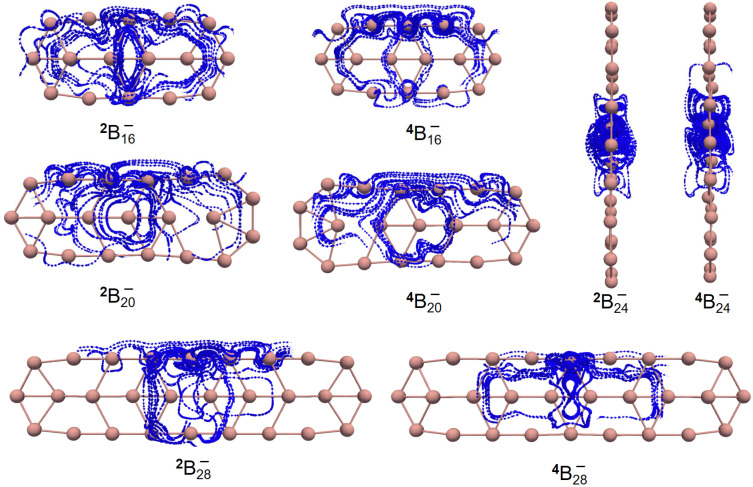
The GIMIC diagrams of both the ground and excited state of $B_{16}^{-}$, $B_{20}^{-}$, $B_{24}^{-}$, and $B_{28}^{-}$. Molecular renderings were achieved via the ParaView [63] software (v. 4).

**Figure 5:**
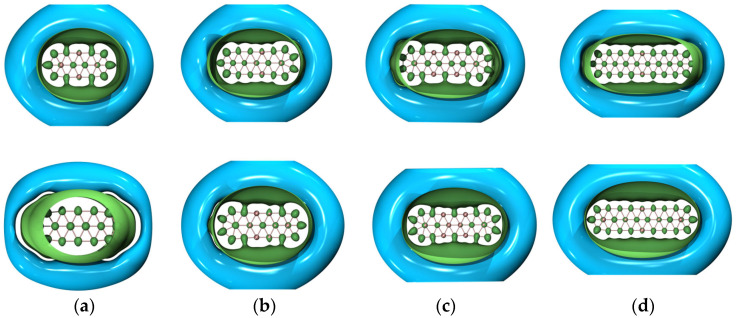
Three-dimensional ICSS maps of both ground- (upper panel) and excited-state (lower panel) (**a**) $B_{16}^{-}$, (**b**) $B_{20}^{-}$, (**c**) $B_{24}^{-}$, and (**d**) $B_{28}^{-}$. The isovalue was set to be 0.02 a.u. The inner green surface is positive and the outer cyan surface is negative. Molecular renderings were achieved via the VMD 1.9.4 [55] software.

Figure 4 shows the GIMIC (gauge-including magnetically induced current) [59,60] distributions of both ground- and excited-state $B_{16}^{-}$, $B_{20}^{-}$, $B_{24}^{-}$ and $B_{28}^{-}$. For ^2^$B_{16}^{-}$ and ^4^$B_{16}^{-}$, the induced electric currents are running counter-clockwise, which is indicative of aromaticity, as evidenced by the negative NICS (nucleus-independent chemical shift) [61] values as shown in Table 2. Similar results are observed for $B_{24}^{-}$ and $B_{28}^{-}$. However, this is not the case for $B_{20}^{-}$. The overall effect indicates that $B_{20}^{-}$ is antiaromatic while the *Z*-component of the induced electric current also runs in a counter-clockwise manner as showcased by the NICS_ZZ_ values in Table 2. Yet, the dominant contributions of the induced electric current lie in the *x*–*y*-plane, which is the source of antiaromaticity. To go a step further, Figure 5 showcases the 3D isotropic shielding surface (ICSS) [62] calculations for both ground- and excited-state boron clusters and it is clearly revealed that there exists a strongly shielded chemical environment along the direction perpendicular to the quasi-planar boron clusters.

Hyperfine coupling constants [64,65] provide a direct experimental measure of the distribution of unpaired spin density in paramagnetic molecules. The interactions of unpaired electrons with external magnetic fields arise from the Zeeman effect and from the hyperfine coupling with nuclei having nonzero spins. The latter contribution is related to the chemical environment. For each nucleus N of a molecule located at $r_{N}$, the isotropic component of the hyperfine interaction tensor, a(N), is related to the local spin density through [66]

$$a\left(N\right)=\frac{8\pi }{3}\beta_{e}\beta_{N}g_{N}\sum_{\mu \nu }P_{\mu \nu }^{\alpha -\beta }\left\langle \varphi_{\mu }|\delta (r-r_{N})|\varphi_{\nu }\right\rangle$$

where $\beta_{e}$, $\beta_{N}$, and $g_{N}$ are the electronic and nuclear magnetons and the nuclear magnetogiric ratio, the indices μ and ν run over the basis functions, $P_{\mu \nu }^{\alpha -\beta }$ is the difference between the density matrices of spin α and spin β electrons, and $\delta (r-r_{N})$ is the Dirac delta function. Therefore, once the density matrices for different spins have been determined, the calculation of a(N) for each nucleus is achieved in a straightforward way. The (isotropic) hyperfine coupling tensor, $A_{iso}^{N}$, consists of the Fermi contact term ($A_{FC}^{N}$) and a spin orbit correction, the pseudocontact term ($A_{PC}^{N}$).

Shown in Table 3 are the isotropic NMR shielding (α_iso_) constants and hyperfine coupling (**A**_iso_) constants for both ground- and excited-state $B_{16}^{-}$ at the PBE0/pcJ-2 [67,68] level. It is clearly shown that the 16 boron atoms can be roughly grouped into 5 different atoms in different chemical environments as evidenced by both the α_iso_ and **A**_iso_ data. Among all the boron atoms, one can easily see that atoms 5 and 6 (as shown in Scheme 1), lying at the two ends of the middle line composed of atoms 1–6, are the most unique. For example, they have the least positive α_iso_ values and the most negative **A**_iso_ data. In addition, they undergo the largest changes when going from the ground state to the excited state. Specifically, **A**_iso_ changes by ~18 MHz while the largest change of the other atoms is ~8 MHz. Similar trends can be observed for $B_{20}^{-}$, $B_{24}^{-}$, and $B_{28}^{-}$ as shown in Table 4, Table 5 and Table 6.

## 3. Discussion

**Figure 6:**
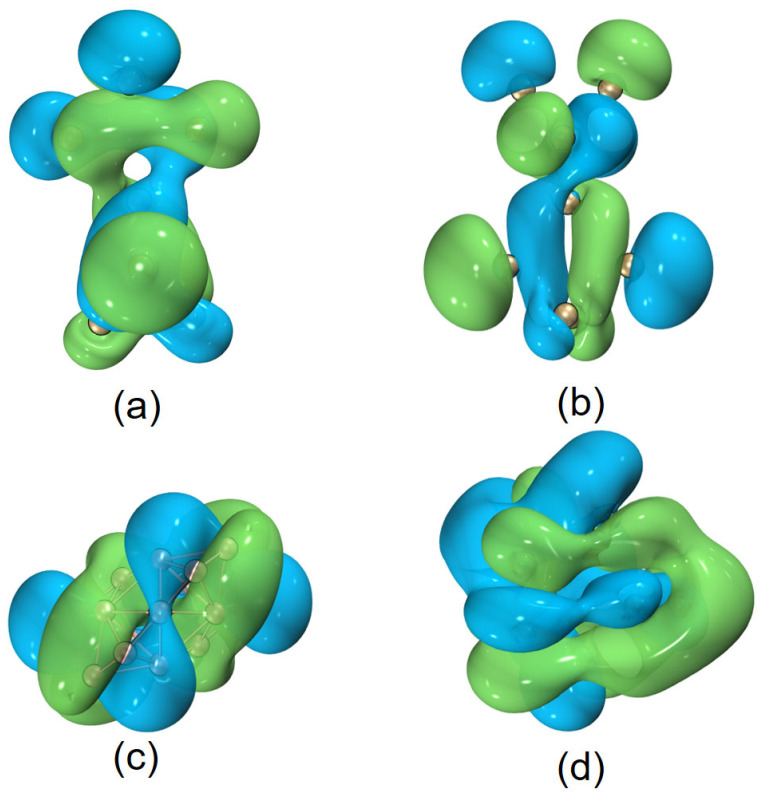
Helical HOMOs of [(**a**,**b**)] $P_{9}^{+}$, (**c**) Be_6_$B_{11}^{-}$, and (**d**) ${\mathrm{As}}_{11}^{3-}$, respectively. The isovalue was set to be 0.02 a.u. for $P_{9}^{+}$ and 0.01 a.u. for Be_6_$B_{11}^{-}$ and ${\mathrm{As}}_{11}^{3-}$. Molecular renderings were achieved via the VMD 1.9.4 [55] software.

Helical frontier molecular orbitals were reported first for hydrocarbon systems, then also for boron-containing molecules [10]. In this work, we have also observed similar results for quasi-planar boron clusters. In addition, as shown in Figure 6, for some other helical (inorganic) motifs, $P_{9}^{+}$, Be_6_$B_{11}^{-}$ (a B_11_ helical structure plus a distorted prism of Be_6_), and ${\mathrm{As}}_{11}^{3-}$, helical frontier molecular orbitals are also observed. Is this helicity a ubiquitous phenomenon or a special feature of some elements in special molecular topologies? This seems to be an open question, and will be a topic for future research.

**Figure 7:**
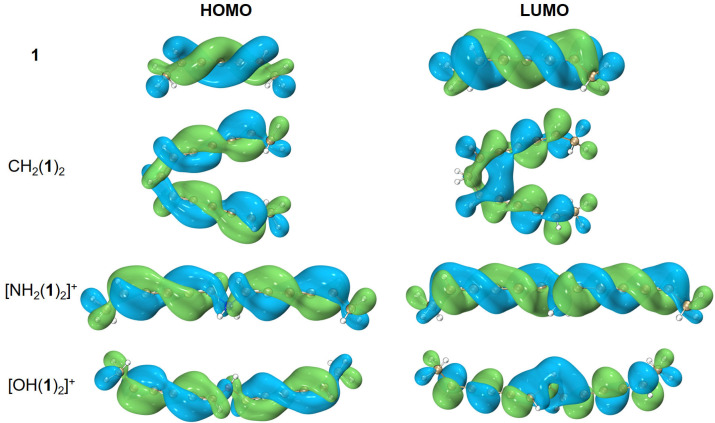
Elongation/enhancement in helical frontier molecular orbitals. The isovalue was set to be 0.02 a.u. Molecular renderings were achieved via the VMD 1.9.4 [55] software.

While a systematic rule for designing molecular templates with helical spin densities is unknown to us, we can show how to elongate the helical frontier molecular orbitals from a given template structure. For example, starting from one conjugated hydrocarbon molecule **1** with helical frontier molecular orbitals, combining two monomers of **1** and a linker, such as CH_2_(**1**)_2_, [NH_2_(**1**)_2_]^+^, and [OH(**1**)_2_]^+^, leads to elongated or enlarged helical frontier molecular orbitals as shown in Figure 7. Yet, when three or four monomers of **1** are grouped together, such as CH(**1**)_3_ or C(**1**)_4_, the helical frontier molecular orbitals are no longer elongated (results not shown). Is it possible to generate an infinite chain of (**1**)_∞_? We do not know; possibly other linkers would work better. For the anionic boron clusters, we failed to even generate a dimer of $B_{16}^{-}$; this is presumably because the repulsion between the anionic monomers prevents electron delocalization between them.

Finally, we have to point out that in a broader sense, the dissection of chiral boron clusters and the electron spin should be beneficial to its applications to chiral spintronics and materials [69,70,71].

## 4. Materials and Methods

To obtain the MPP or SDP values for a molecule, the least square method is used to generate a fitted plane by all the atoms considered. First, one can have a coordinate matrix whose dimension is 3 × *N*_atom_. After subtracting out the geometry center, one can easily perform a singular value decomposition for this matrix. To obtain the coefficients (*A*, *B*, and *C*) and constant (*D*) for a fitting plane *Ax* + *By* + *Cz* + *D* = 0, one can set *A* = *u*_1_, *B* = *u*_2_, *C* = *u*_3_ to be the left singular vectors corresponding to the least singular values. The constant *D* is determined to be −(*u*_1_*x*_c_ + *u*_2_*y*_c_ + *u*_3_*z*_c_) if the fitting plane passes through the geometry center (*x*_c_, *y*_c_, *z*_c_) of a molecule. The molecular planarity parameter (MPP) can be readily calculated as the root-mean-squared deviation (RMSD) of the atoms from the fitting plane, $MPP=\sqrt{\frac{1}{N_{atom}}\sum_{i}d_{i}^{2}}$, where $d_{i}$ is the distance between an atom *i* and the fitting plane, and it can be easily evaluated as $d_{i}=\frac{\left|Ax_{i}+By_{i}+Cz_{i}+D\right|}{\sqrt{A^{2}+B^{2}+C^{2}}}\;$. The *signed* distance of an atom *i* to the fitting plane is defined as $d_{i}^{s}=\frac{Ax_{i}+By_{i}+Cz_{i}+D}{\sqrt{A^{2}+B^{2}+C^{2}}}$; then, the span of deviation from the plane (SDP) can be calculated as $SDP=d_{max}^{s}-d_{min}^{s}$, where $d_{max}^{s}$/$d_{min}^{s}$ denote the most positive/negative values of $d^{s}$ among all considered atoms, respectively.

For all the molecular systems, structure optimization was performed at the density functional theory (DFT) [72,73] PBE0/6-311+G(d) level. Stability of molecular wavefunctions was confirmed via keywords of “guess = mix” and “stable = opt” in Gaussian 16 [74]. Vibrational frequency calculations were ensued to make sure that all the structures were true local minima on the potential energy surface. The optimized atomic Cartesian coordinates are supplied in the Supplementary Materials. Multireference (MR) characteristics of all boron clusters were checked via the T1 diagnostics [75] regarding the coupled cluster theory with single and double substitutions [CCSD/6-311+G(d)] and the frozen core formalism was used for CCSD calculations. The reported values for $B_{16}^{-}$, $B_{20}^{-}$, $B_{24}^{-}$, and $B_{28}^{-}$ are 0.0365, 0.0495, 0.0344, and 0.0411 for the ground state, and 0.0414, 0.0408, 0.0398, and 0.0367 for the excited state, indicative of non-negligible multi-reference characteristics (because their T1 > 0.02). For the ground state, we also employed a larger basis set cc-pVTZ [76] and very close results were obtained for T1, 0.0360, 0.0488, 0.0344, and 0.0415, respectively. However, for radicals, somewhat larger T1 values (~0.03) are acceptable, and the usual thresholds for T1 are based on accurate quantitative recovery of the dynamic correlation energy, rather than qualitative considerations like those we consider here. The T1 values we report are consistent with the coefficient of the leading determinant being greater than 0.90, implying that single-determinant methods (DFT) and single-reference methods (UCCSD) are good enough to elucidate qualitative features of the boron clusters. 

In addition, to force all the atoms to be exactly in a plane, we simply set, for example, the *z*-components of all the boron atoms to be zero if the original quasi-planar boron cluster is lying in a *xy*-plane.

To further analyze the aromaticity properties of boron clusters, we employed PBE0/pcJ-2 to calculate the global NICS (nucleus-independent chemical shift) values and GIMIC (gauge-including magnetically induced current) distributions. NMR chemical shielding constants and isotropic hyperfine coupling parameters were obtained at the PBE0/pcJ-2 level with default gauge-including atomic orbitals (GIAOs) [77,78,79,80,81]. The global NICS value is obtained at the geometric center, denoted as NICS(0), and its *z*-axis component as NICS(0)_ZZ_. Moreover, another two points (1 Å away from a global center) are also considered and they are signified as NICS(1) and NICS(−1), with their *z*-axis components denoted as NICS(1)_ZZ_ and NICS(−1)_ZZ_.

All DFT calculations were performed by using the Gaussian 16 package with tight self-consistent field (SCF) convergence criteria and ultrafine integration grids to ensure good accuracy. Multiwfn 3.8 [82] software was used to analyze the planarity of boron clusters and prepare the ICSS input files.

## 5. Summary

We have observed that helical molecular orbitals and helical spin densities can coexist in a chiral quasi-planar boron cluster. We show that this intriguing phenomenon emerges due to the chiral Jahn–Teller distortion of planar boron clusters and show how to generate elongated or enhanced helical molecular orbitals by grouping building blocks together via a linker. Finally, we found that helical inorganic species have a strong propensity to assume helical molecular orbitals. For potential applications in spintronics, it is interesting to study whether the observed helical spin densities would still be observed for boron clusters adsorbed on metal surfaces. Work along these lines is in progress and the results will be presented elsewhere.

## Figures and Tables

**Table 1:** Molecular planarity parameter (MPP), span of deviation from the plane (SDP), maximum positive/negative deviation (MPD/MND) to the fitted plane *^a^*.

Species	MPP	SDP	MPD	MND
^2^ $B_{16}^{-}$	0.090	0.442	0.221	−0.221
^4^ $B_{16}^{-}$	0.017	0.091	0.047	−0.044
^2^ $B_{20}^{-}$	0.081	0.382	0.263	−0.119
^4^ $B_{20}^{-}$	0.083	0.322	0.179	−0.143
^2^ $B_{24}^{-}$	0.021	0.074	0.037	−0.037
^4^ $B_{24}^{-}$	0.039	0.196	0.098	−0.098
^2^ $B_{28}^{-}$	0.092	0.440	0.302	−0.138
^4^ $B_{28}^{-}$	0.103	0.404	0.212	−0.192

*^a^* Units are in Å.

**Table 2:** Ground- and excited-state aromaticity of $B_{16}^{-}$, $B_{20}^{-}$, $B_{24}^{-}$, and $B_{28}^{-}$ in terms of the global NICS values *^a^*.

Species	NICS(0)	NICS(0)_ZZ_	NICS(1)	NICS(1)_ZZ_	NICS(−1)	NICS(−1)_ZZ_
^2^ $B_{16}^{-}$	−16.67	−48.74	−17.90	−39.52	−17.90	−39.52
^4^ $B_{16}^{-}$	−16.76	−111.92	−5.85	−4.66	−5.83	−4.66
^2^ $B_{20}^{-}$	15.51	−28.62	−15.89	−31.60	−11.26	−23.02
^4^ $B_{20}^{-}$	22.94	−15.48	−13.61	−32.13	−13.57	−30.98
^2^ $B_{24}^{-}$	−29.53	−47.96	−14.42	−26.37	−14.42	−26.37
^4^ $B_{24}^{-}$	−26.90	−59.08	−16.85	−38.44	−16.85	−38.44
^2^ $B_{28}^{-}$	−18.76	−33.63	−8.27	−16.35	−8.87	−15.98
^4^ $B_{28}^{-}$	−17.24	−36.36	−11.94	−22.62	−11.74	−23.33

*^a^* PBE0/pcJ-2. Units are in ppm. NICS(0) corresponds to the NICS value at the global geometric center, NICS(1) at point 1 Å above the global geometric center, and NICS(−1) at point 1.0 Å below the global geometric center. The *z*-axis component is denoted as NICS_ZZ_.

**Table 3:** Isotropic NMR shielding constants (α_iso_, in ppm) and Fermi contact couplings (**A**_iso_, in MHz) for ground-state (columns 2, 3, 7, and 8) and excited-state (columns 4, 5, 9, and 10) $B_{16}^{-}$.

no.	^2^α_iso_	^2^A_iso_	^4^α_iso_	^4^A_iso_	no.	^2^α_iso_	^2^A_iso_	^4^α_iso_	^4^A_iso_
1	77.66	−2.98	78.50	−1.53	9	35.33	40.64	25.78	32.87
2	77.66	−2.98	78.50	−1.56	10	35.34	40.58	25.78	32.86
3	81.47	−5.77	72.32	−8.74	11	69.43	−11.44	82.41	−10.30
4	81.47	−5.77	72.33	−8.74	12	69.42	−11.46	82.41	−10.30
5	8.77	−35.62	36.11	−17.18	13	69.43	−11.44	82.41	−10.30
6	8.77	−35.62	36.11	−17.19	14	69.43	−11.46	82.41	−10.30
7	35.33	40.66	25.78	32.86	15	102.86	−3.11	78.37	0.72
8	35.34	40.60	25.78	32.85	16	102.86	−3.10	78.37	0.72

**Table 4:** Isotropic NMR shielding constants (α_iso_, in ppm) and Fermi contact couplings (**A**_iso_, in MHz) for ground-state (columns 2, 3, 7, and 8) and excited-state (columns 4, 5, 9, and 10) $B_{20}^{-}$.

no.	^2^α_iso_	^2^A_iso_	^4^α_iso_	^4^A_iso_	no.	^2^α_iso_	^2^A_iso_	^4^α_iso_	^4^A_iso_
1	82.41	0.71	92.94	1.07	11	39.89	24.08	44.65	13.31
2	75.99	−17.57	64.17	−7.19	12	39.89	24.09	44.53	13.25
3	94.80	5.82	117.43	−0.87	13	26.91	−9.85	30.45	−7.18
4	82.47	−15.30	78.11	−12.48	14	26.91	−9.86	30.46	−7.30
5	18.41	35.48	38.64	35.92	15	78.73	2.80	92.78	2.47
6	78.05	−9.61	74.29	−9.04	16	81.60	−10.16	69.88	−8.16
7	81.60	−10.16	69.82	−8.20	17	69.79	−9.31	79.02	−7.43
8	18.42	35.48	38.84	35.95	18	94.79	5.81	117.62	−0.91
9	34.65	−21.98	19.20	−27.86	19	77.81	4.31	86.55	0.07
10	82.41	0.73	93.02	1.02	20	75.99	−17.58	64.29	−7.26

**Table 5:** Isotropic NMR shielding constants (α_iso_, in ppm) and Fermi contact couplings (**A**_iso_, in MHz) for ground-state (columns 2, 3, 7, and 8) and excited-state (columns 4, 5, 9, and 10) $B_{24}^{-}$.

no.	^2^α_iso_	^2^A_iso_	^4^α_iso_	^4^A_iso_	no.	^2^α_iso_	^2^A_iso_	^4^α_iso_	^4^A_iso_
1	80.03	−5.46	79.50	−7.42	13	118.46	−9.58	120.01	−3.83
2	78.34	1.45	79.92	5.62	14	118.46	−9.58	120.01	−3.83
3	83.41	−5.35	88.96	−3.69	15	118.46	−9.58	120.01	−3.83
4	83.41	−5.35	88.96	−3.69	16	68.85	12.02	70.01	4.22
5	78.34	1.45	79.92	5.62	17	68.85	12.02	70.01	4.22
6	80.03	−5.46	79.50	−7.42	18	28.82	−8.41	17.95	−3.84
7	38.44	8.36	45.29	1.03	19	28.82	−8.41	17.95	−3.84
8	66.10	−7.13	64.54	−4.36	20	38.44	8.36	45.29	1.03
9	66.10	−7.13	64.54	−4.36	21	38.44	8.36	45.29	1.03
10	66.10	−7.13	64.54	−4.36	22	28.82	−8.41	17.95	−3.84
11	66.10	−7.13	64.54	−4.36	23	28.82	−8.41	17.95	−3.84
12	118.46	−9.58	120.01	−3.83	24	38.44	8.36	45.29	1.03

**Table 6:** Isotropic NMR shielding constants (α_iso_, in ppm) and Fermi contact couplings (**A**_iso_, in MHz) for ground-state (columns 2, 3, 7, and 8) and excited-state (columns 4, 5, 9, and 10) $B_{28}^{-}$.

no.	^2^α_iso_	^2^A_iso_	^4^α_iso_	^4^A_iso_	no.	^2^α_iso_	^2^A_iso_	^4^α_iso_	^4^A_iso_
1	79.30	−7.09	70.30	5.31	15	69.93	−19.78	84.30	0.54
2	73.04	−1.60	84.98	−5.58	16	96.89	31.15	98.54	−1.44
3	83.12	−5.16	78.07	−0.08	17	96.89	31.14	98.57	−1.44
4	84.30	−6.42	78.12	−0.08	18	78.19	−18.49	81.49	−5.32
5	76.95	−3.58	84.99	−5.61	19	74.86	−16.35	81.54	−5.27
6	79.95	−6.84	70.29	5.14	20	74.86	−16.35	81.52	−5.26
7	78.19	−18.49	81.52	−5.34	21	70.58	−4.58	75.02	−5.81
8	82.74	13.22	91.03	−1.62	22	17.52	50.44	29.89	11.67
9	82.74	13.22	90.88	−1.57	23	17.52	50.45	29.91	11.70
10	77.86	17.29	90.69	−1.61	24	37.06	−33.92	16.67	−9.92
11	77.87	17.30	90.80	−1.66	25	63.54	−0.51	74.89	−5.83
12	68.99	−21.28	84.21	0.63	26	42.21	−19.68	16.75	−9.76
13	68.99	−21.28	84.33	0.62	27	14.01	33.86	29.71	11.46
14	69.93	−19.77	84.19	0.55	28	14.00	33.88	29.66	11.45

## Data Availability

Data are contained within the article.

## Supplementary Materials

The following supporting information can be downloaded at: https://www.mdpi.com/article/10.3390/molecules29071624/s1, Optimized structures, excited-state VCD spectra, excited-state spin densities, fitted parameters of the planes of boron clusters, and the ground-state spin density maps of planar boron clusters.
