# Supplementary material for: Chiral Jahn–Teller Distortion in Quasi-Planar Boron Clusters
Source: Molecules. 2024 Apr 4;29(7):1624. doi: 10.3390/molecules29071624 (PMC11013085; doi:10.3390/molecules29071624)
Supplement: Supplementary file 1 [file molecules-29-01624-s001.zip › molecules-2890128-supplementary.pdf]

Supplementary information for

Chiral Jahn-Teller Distortion in Quasi-Planar Boron Clusters

Dongbo Zhao[1,*], Yilin Zhao[2], Tianlv Xu[3], Xin He[4], Shankai Hu[1], Paul W. Ayers[2,*] and Shubin Liu[5,6,*]

[1]Institute of Biomedical Research, Yunnan University, Kunming 650500, Yunnan, China
[2]Department of Chemistry and Chemical Biology, McMaster University, Hamilton, ONL8S 4M1 Canada
[3]College of Chemistry and Chemical Engineering, Hunan Normal University, Changsha, Hunan 410081, China
[4]Qingdao Institute for Theoretical and Computational Sciences, Shandong University, Qingdao 266237, China
[5]Research Computing Center, University of North Carolina, Chapel Hill, North Carolina 27599-3420, USA
[6]Department of Chemistry, University of North Carolina, Chapel Hill, North Carolina 27599-3290, USA
*Correspondence:
dongbo@ynu.edu.cn (D.Z.);
ayers@mcmaster.ca (P.W.A.);
shubin@email.unc.edu (S.L.).

Table S1. The coefficients of the fitted planes.

species	A	B	C	D
$^{2}B_{16}^{-}$	0.00000	0.00000	1.00000	0.00000
$^{2}B_{20}^{-}$	0.00000	0.00000	1.00000	0.00000
$^{2}B_{24}^{-}$	–0.99999	–0.00378	–0.00000	–0.00000
$^{2}B_{28}^{-}$	0.00000	0.00000	1.00000	–0.00000
$^{4}B_{16}^{-}$	0.00000	–0.00000	1.00000	0.00000
$^{4}B_{20}^{-}$	0.00000	–0.00000	1.00000	0.00000
$^{4}B_{24}^{-}$	1.00000	–0.00275	0.00000	–0.00000
$^{4}B_{28}^{-}$	–0.00001	0.00006	–1.00000	0.00000

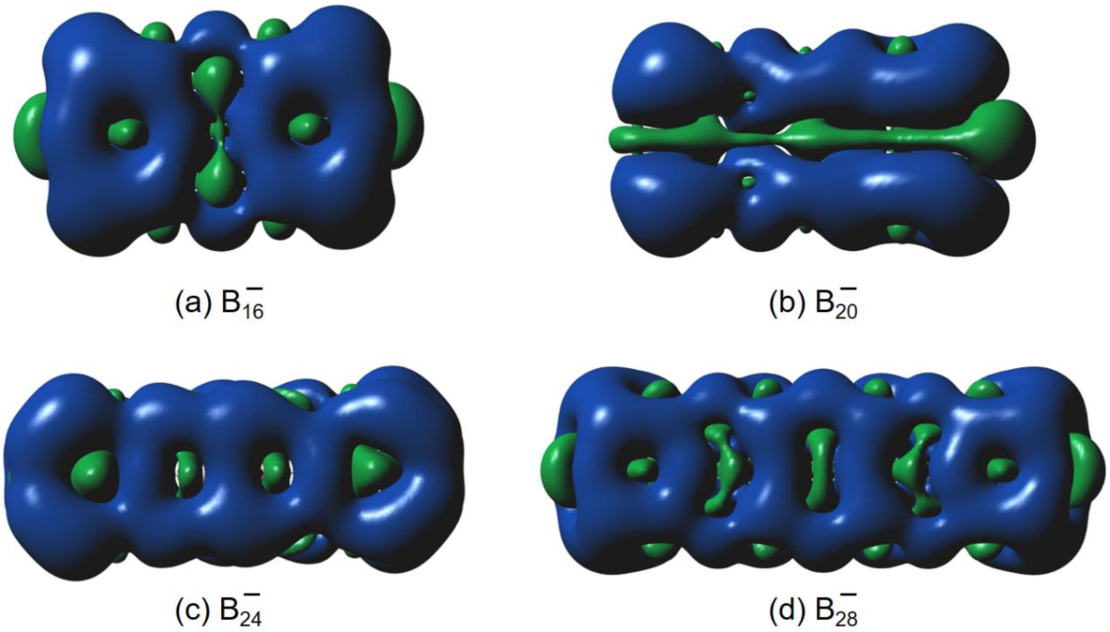

Figure S1. Shown here are the spin density maps of excited-state (a) $^4B_{16}^-$, (b) $^4B_{20}^-$, (c) $^4B_{24}^-$, and (d) $^4B_{28}^-$ obtained at the PBE0/6-311+G(d) level. The isovalue was set to be 0.0004 a.u.

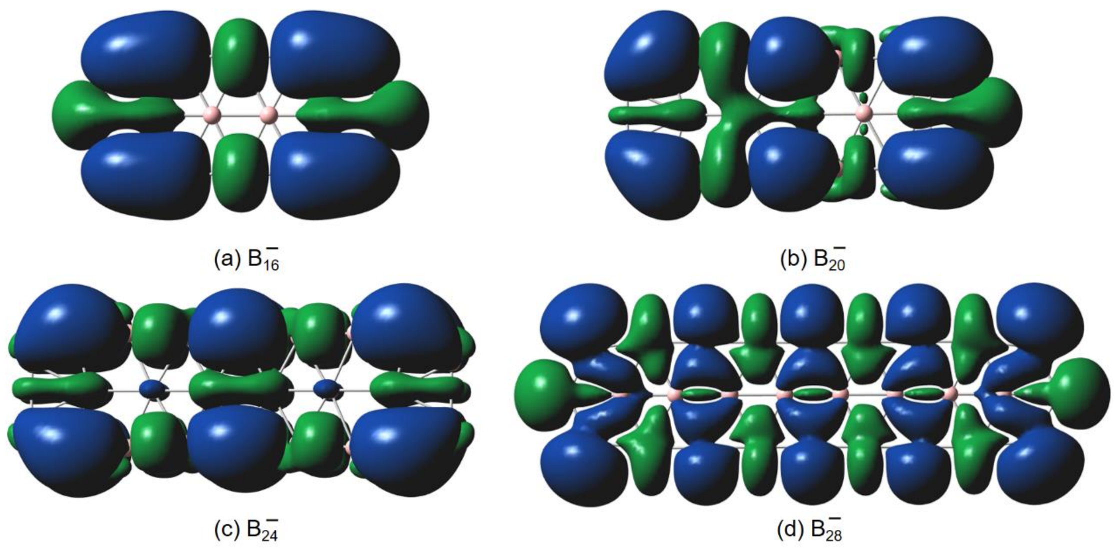

Figure S2. Shown here are the spin density maps of ground-state (a) $^{2}B_{16}^{-}$, (b) $^{2}B_{20}^{-}$, (c) $^{2}B_{24}^{-}$, and (d) $^{2}B_{28}^{-}$ obtained at the PBE0/6-311+G(d) level with boron atoms forced to be in a plane. The isovalue was set to be 0.0004 a.u.

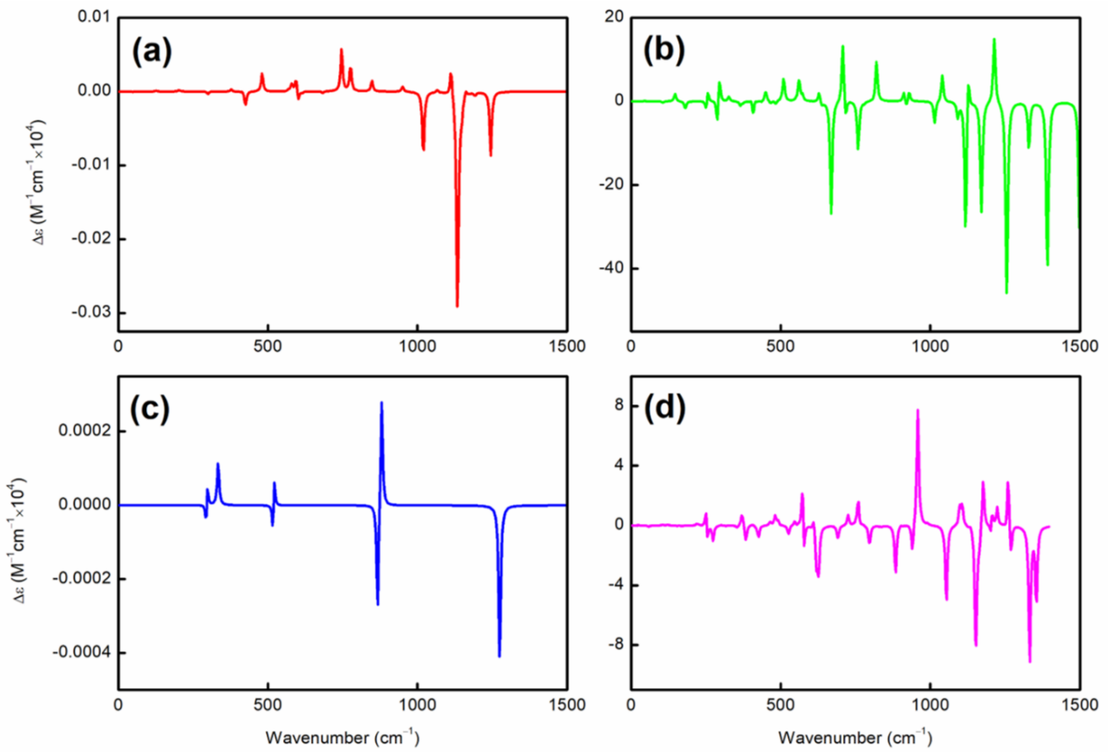

Figure S3. Excited-state VCD spectra of (a) $^4B_{16}^-$, (b) $^4B_{20}^-$, (c) $^4B_{24}^-$, and (d) $^4B_{28}^-$ obtained at the PBE0/6-311+G(d) level. The *x*/*y*-axis spans from (a) to (d).

$^2B_{16}^-$			
B	0.76706500	-0.00001200	0.01605200
B	-0.76706000	0.00001700	-0.01615800
B	2.37466600	-0.00002500	0.22121500
B	-2.37468300	0.00002900	-0.22115200
B	3.96019200	-0.00000900	-0.07694600
B	-3.96018500	0.00001000	0.07709700
B	3.21095700	-1.40875300	-0.05721800
B	3.21097200	1.40874200	-0.05700100
B	-3.21094500	1.40875400	0.05736100
B	-3.21095800	-1.40874200	0.05717500
B	1.63741300	-1.61594400	0.04092400
B	1.63744700	1.61594300	0.04116200
B	-1.63741400	1.61594200	-0.04104800
B	-1.63745900	-1.61595200	-0.04137700
B	0.00003400	1.56311700	-0.00051500
B	-0.00004200	-1.56311700	0.00042900

$^2B_{20}^-$			
B	-1.07844700	-1.54869500	0.02466900
B	2.11279500	-1.59892500	0.02171000
B	0.52838800	1.50991300	-0.01282000
B	-0.26223600	-0.00000700	0.11042100
B	-4.28634900	-1.38518900	-0.11941800
B	3.19474400	-0.00000200	-0.00594300
B	-2.69738600	1.55696800	0.02350400
B	-4.28635600	1.38519000	-0.11940500
B	-5.05518400	0.00000100	-0.08454500
B	-1.07845900	1.54868100	0.02457500
B	3.64586700	-1.75530700	-0.00356300
B	3.64587500	1.75530300	-0.00371100
B	4.80354600	0.77090700	-0.02350100
B	4.80353900	-0.77090900	-0.02324400
B	1.39399200	0.00000200	-0.10212600
B	-2.69737600	-1.55696100	0.02350300

B	-3.46381700	0.00000000	0.26319200
B	0.52838400	-1.50990700	-0.01312500
B	-1.86432600	-0.00000400	-0.00196100
B	2.11280300	1.59894000	0.02178700

**$^2B_{24}^-$**			
B	0.00147700	4.27685400	0.00000000
B	-0.04193200	2.48783100	0.00000000
B	0.03382600	0.81571200	0.00000000
B	-0.03382600	-0.81571200	0.00000000
B	0.04193200	-2.48783100	0.00000000
B	-0.00147700	-4.27685400	0.00000000
B	0.02969600	-4.72724800	1.74871500
B	0.03436800	-3.18798800	-1.60276300
B	0.03436800	-3.18798800	1.60276300
B	-0.03436800	3.18798800	-1.60276300
B	-0.03436800	3.18798800	1.60276300
B	-0.01643000	1.60824700	1.49040200
B	-0.01643000	1.60824700	-1.49040200
B	0.01643000	-1.60824700	-1.49040200
B	0.01643000	-1.60824700	1.49040200
B	0.00000000	0.00000000	-1.52771600
B	0.00000000	0.00000000	1.52771600
B	0.00000000	-5.89379500	0.76474100
B	0.00000000	-5.89379500	-0.76474100
B	0.02969600	-4.72724800	-1.74871500
B	-0.02969600	4.72724800	-1.74871500
B	0.00000000	5.89379500	-0.76474100
B	0.00000000	5.89379500	0.76474100
B	-0.02969600	4.72724800	1.74871500

**$^2B_{28}^-$**			
B	4.01886100	-0.00008000	-0.13718200
B	2.41969300	0.00001300	0.12861200
B	0.82288100	0.00004500	-0.13829500

B	-0.81367400	0.00006000	0.00714500
B	-2.42185300	-0.00000400	0.01169500
B	-4.01398100	0.00000500	-0.13793700
B	-4.85032400	-1.55340300	0.00577500
B	-3.23445000	1.54558900	-0.03831800
B	-3.23443600	-1.54555000	-0.03854300
B	3.23581900	1.54700700	-0.03810600
B	3.23574500	-1.54703300	-0.03777000
B	1.61186400	-1.53721700	-0.05652500
B	1.61189900	1.53731800	-0.05679500
B	-1.60443000	1.53892200	-0.04162700
B	-1.60439300	-1.53879300	-0.04198900
B	0.00350000	1.52939600	-0.05206100
B	0.00351200	-1.52924800	-0.05209800
B	-4.85032200	1.55332700	0.00633300
B	4.84452500	1.54710400	0.01165800
B	4.84447800	-1.54708300	0.01212700
B	-5.59739900	-0.00005600	0.18212100
B	-6.46588100	1.39223800	0.03364800
B	-6.46585300	-1.39232600	0.03317800
B	-7.21668500	-0.00004800	0.09765900
B	5.59347100	0.00001400	0.30182100
B	7.21520300	-0.00004800	0.08255300
B	6.45610600	1.37667300	-0.02382800
B	6.45612300	-1.37682100	-0.02325000

$^4B_{16}^-$			
B	0.79392500	0.00001400	0.04744400
B	-0.79387000	-0.00001400	-0.04440400
B	2.38837300	-0.00000500	-0.01688900
B	-2.38842100	0.00000500	0.01643100
B	4.01377200	0.00000300	-0.00438200
B	-4.01381400	-0.00000200	0.00184900
B	3.26175000	1.39677500	-0.00075500
B	3.26175400	-1.39677400	-0.00074200
B	-3.26176500	-1.39678000	-0.00069000
B	-3.26177100	1.39677900	-0.00073300
B	1.63338200	1.55245700	0.00521000
B	1.63338800	-1.55245700	0.00529900
B	-1.63336300	-1.55246600	-0.00439500
B	-1.63337100	1.55246800	-0.00453300
B	0.00001200	-1.55535200	0.00050600
B	0.00002000	1.55535000	0.00078200

$^4B_{20}^-$			
B	1.07373300	1.53640600	-0.07100500
B	-2.10017800	1.62724900	-0.00369800
B	-0.53584400	-1.52509400	-0.03820100
B	0.25898400	-0.00043800	-0.03762100
B	4.31917200	1.40714600	0.11120200
B	-3.22526500	0.00020200	0.04530100
B	2.71546900	-1.57122200	-0.01288100
B	4.31845500	-1.40679100	0.12323200
B	5.05770000	0.00011600	0.17887500
B	1.07390200	-1.53622100	-0.06000300
B	-3.65974400	1.75116000	0.03707200
B	-3.66009700	-1.75062000	0.01325700
B	-4.81847300	-0.77210800	0.07709200
B	-4.81827100	0.77193800	0.08787200
B	-1.40987700	0.00009100	-0.07990800
B	2.71602400	1.57099700	-0.02525300

B	3.48066900	-0.00032100	-0.13618500
B	-0.53535700	1.52542600	-0.04951100
B	1.84941800	-0.00083500	-0.14347900
B	-2.10042100	-1.62707900	-0.01616000

**$^4B_{24}^-$**			
B	0.00656100	4.26979600	0.00000000
B	-0.09142000	2.47428100	0.00000000
B	0.07134500	0.83334000	0.00000000
B	-0.07134500	-0.83334000	0.00000000
B	0.09142000	-2.47428100	0.00000000
B	-0.00656100	-4.26979600	0.00000000
B	-0.02774400	-4.71810400	1.75756400
B	-0.04559800	-3.17106000	-1.64607500
B	-0.04559800	-3.17106000	1.64607500
B	0.04559800	3.17106000	-1.64607500
B	0.04559800	3.17106000	1.64607500
B	0.01169800	1.60200900	1.50465700
B	0.01169800	1.60200900	-1.50465700
B	-0.01169800	-1.60200900	-1.50465700
B	-0.01169800	-1.60200900	1.50465700
B	0.00000000	0.00000000	-1.55079700
B	0.00000000	0.00000000	1.55079700
B	0.00000000	-5.87439100	0.77736900
B	0.00000000	-5.87439100	-0.77736900
B	-0.02774400	-4.71810400	-1.75756400
B	0.02774400	4.71810400	-1.75756400
B	0.00000000	5.87439100	-0.77736900
B	0.00000000	5.87439100	0.77736900
B	0.02774400	4.71810400	1.75756400

**$^4B_{28}^-$**			
B	-4.00448800	-0.00003700	-0.00691000
B	-2.42460400	0.00074100	-0.12150100
B	-0.80386200	0.00007800	0.02931900

B	0.80340800	-0.00033400	0.03028600
B	2.42428700	-0.00043300	-0.11378600
B	4.00406300	-0.00016600	-0.00603600
B	4.85282100	-1.57091800	-0.00387200
B	3.21401200	1.55435800	-0.03699300
B	3.21402500	-1.55540000	-0.02928600
B	-3.21407600	1.55630700	-0.03482900
B	-3.21408300	-1.55527700	-0.04368800
B	-1.61371600	-1.57277700	-0.03881000
B	-1.61348000	1.57273500	-0.03485900
B	1.61337800	1.57403800	-0.03287000
B	1.61330300	-1.57331100	-0.02893800
B	0.00006900	1.53317600	-0.03583500
B	-0.00026200	-1.53276900	-0.03553200
B	4.85302300	1.57077500	-0.01376700
B	-4.85270800	1.57027800	-0.00493600
B	-4.85281000	-1.57026200	-0.01528100
B	5.60423900	-0.00091000	-0.21048800
B	6.43051200	1.39738600	0.14862600
B	6.43028500	-1.39682600	0.15835700
B	7.18819400	0.00038800	0.18541700
B	-5.60493200	0.00082900	-0.21174100
B	-7.18744500	-0.00066000	0.19187900
B	-6.42950700	1.39633400	0.16312600
B	-6.42964700	-1.39734800	0.15294900
